# Modulation of endothelial cell migration by ER stress and insulin resistance: a role during maternal obesity?

**DOI:** 10.3389/fphar.2014.00189

**Published:** 2014-08-19

**Authors:** Pablo J. Sáez, Roberto Villalobos-Labra, Francisco Westermeier, Luis Sobrevia, Marcelo Farías-Jofré

**Affiliations:** ^1^Cellular and Molecular Physiology Laboratory, Division of Obstetrics and Gynecology, School of Medicine, Faculty of Medicine, Pontificia Universidad Católica de ChileSantiago, Chile; ^2^Facultad de Ciencia, Universidad San SebastiánSantiago, Chile; ^3^Advanced Center for Chronic Diseases, Faculty of Chemical and Pharmaceutical Sciences and Faculty of Medicine, University of ChileSantiago, Chile; ^4^University of Queensland Centre for Clinical Research, Faculty of Medicine and Biomedical Sciences, University of QueenslandHerston, QL, Australia; ^5^Faculty of Pharmacy, Universidad de SevillaSevilla, Spain

**Keywords:** mesenchymal migration, unfolded protein response, RhoA, Akt, Scrib, polarization, cytoskeleton

## Abstract

Adverse microenvironmental stimuli can trigger the endoplasmic reticulum (ER) stress pathway, which initiates the unfolded protein response (UPR), to restore protein-folding homeostasis. Several studies show induction of ER stress during obesity. Chronic UPR has been linked to different mechanisms of disease in obese and diabetic individuals, including insulin resistance (IR) and impaired angiogenesis. Endothelial cell (EC) migration is an initial step for angiogenesis, which is associated with remodeling of existing blood vessels. EC migration occurs according to the leader–follower model, involving coordinated processes of chemotaxis, haptotaxis, and mechanotaxis. Thus, a fine-tuning of EC migration is necessary to provide the right timing to form the required vessels during angiogenesis. ER stress modulates EC migration at different levels, usually impairing migration and angiogenesis, although different effects may be observed depending on the tissue and/or microenvironment. In the context of pregnancy, maternal obesity (MO) induces IR in the offspring. Interestingly, several proteins associated with obesity-induced IR are also involved in EC migration, providing a potential link with the ER stress-dependent alterations observed in obese individuals. Different signaling cascades that converge on cytoskeleton regulation directly impact EC migration, including the Akt and/or RhoA pathways. In addition, ER is the main intracellular reservoir for Ca^2+^, which plays a pivotal role during EC migration. Therefore, ER stress-related alterations in Ca^2+^ signaling or Ca^2+^ levels might also produce distorted EC migration. However, the above findings have been studied in the context of adult obesity, and no information has been reported regarding the effect of MO on fetal EC migration. Here we summarize the state of knowledge about the possible mechanisms by which ER stress and IR might impact EC migration and angiogenesis in fetal endothelium exposed to MO during pregnancy.

## INTRODUCTION

Endoplasmic reticulum is the major subcellular membrane organelle, playing a pivotal role in synthesis, folding and maturation of proteins, and providing the main Ca^2+^ reservoir inside the cell (Berridge et al., 2003; Cnop et al., 2012; Hetz et al., 2013). Under certain conditions, the environment induces ER stress and further activation of the UPR, which triggers a cascade of signaling events to restore protein-folding homeostasis (Kozutsumi et al., 1988). This cellular condition, known as ER stress, is induced by different types of stimuli, such as accumulation of unfolded proteins, fatty acids, cytokines, redox state dysregulation, and increased intracellular Ca^2+^ levels (Kozutsumi et al., 1988; Hotamisligil, 2010; Cnop et al., 2012; Fu et al., 2012; Garg et al., 2012; Hetz et al., 2013). Moreover, ER stress is linked to different diseases, including cancer, type II diabetes, and obesity (Hotamisligil, 2010; Cnop et al., 2012; Hetz et al., 2013). Importantly, most if not all of these pathologies are associated with vascular pathologies such as distorted angiogenesis or endothelial dysfunction (Minamino and Kitakaze, 2010; Basha et al., 2012). By affecting EC physiology, ER stress contributes to the vascular dysfunction observed in diabetic retinopathy, cancer, obesity, atherosclerosis, and ischemia (Amin et al., 2012; Hetz et al., 2013; Zeng et al., 2013; Paridaens et al., 2014). One of the most relevant functions of EC is angiogenesis, which is the capacity to form new capillary vessels (Lamalice et al., 2007). Interestingly, ER stress affects two of the basic mechanisms that contribute to angiogenesis (Lamalice et al., 2007): VEGF signaling, and EC migration (Iwawaki et al., 2009; Ghosh et al., 2010; Pereira et al., 2010; Banerjee et al., 2011; Zeng et al., 2013; Paridaens et al., 2014). However, obesity might impact EC migration directly through ER stress and induced IR; Westermeier et al., 2014), because several of the involved proteins, such as RhoA and Akt (also called protein kinase B), also modulate EC migration (Lamalice et al., 2007).

As expected, the development of obesity in adults produces altered angiogenic responses in adipose tissue (Christiaens and Lijnen, 2010). However, in the context of pregnancy, MO not only affects the mother but also can permanently damage fetal tissues [American College of Obstetricians and Gynecologists (ACOG), 2005]. Thus, the adverse intrauterine environment in MO pregnancies could modulate offspring physiology (Bruyndonckx et al., 2013), leading to *in utero* development of IR (Catalano et al., 2009), which ultimately might affect EC migration and angiogenesis. In support of this notion, MO is associated with alterations in serum levels of angiogenic markers (Zera et al., 2014) and changes in VEGF receptor expression patterns in the placenta (Dubova et al., 2011; Saben et al., 2014). Interestingly, very recent studies show that MO induces ER stress in offspring in murine models (Melo et al., 2014; Wu et al., 2014), suggesting that distortions in EC migration and angiogenesis might occur. Since cell migration commands angiogenesis, our goal is to give an integrative overview of how MO-induced ER stress and IR might affect the migratory potential of EC and hence angiogenesis in the offspring, with deleterious consequences for the offspring’s development.

### ER STRESS AND THE UNFOLDED PROTEIN RESPONSE

Multiple environmental stimuli are capable of triggering ER stress (Schroder and Kaufman, 2005). There are three major sensors of ER stress, all of which are ER membrane-associated proteins: ATF6 (α and β isoforms), PERK, and IRE1 (Hetz et al., 2013). While activation of both PERK and IRE1 involves dimerization and phosphorylation, ATF6 activation requires its cleavage and translocation to the nucleus (Hetz et al., 2013). These three pathways interact and produce ER-to-nucleus signaling that reduces protein translation and increases folding capacity (Hotamisligil, 2010). However, differential activation of ER sensors may occur depending on the type and timing of the ER stressor signal (Wu et al., 2007; Fu et al., 2012). The latter will produce different UPR profiles, associated with the specific stimuli triggering the ER stress, the affected cell type(s), and the microenvironment background. In addition, acute versus chronic ER stress may lead to different cellular responses (Wu et al., 2007; Fu et al., 2012).

### ROLE OF ER STRESS IN CELL MIGRATION

The role of ER stress on cell migration and angiogenesis has been studied mainly in cancer cells (Hetz et al., 2013). Several ER stress-related proteins contribute to cell migration and/or angiogenesis in tumors (Paridaens et al., 2014). The three abovementioned ER stress branches usually contribute to angiogenesis in the tumoral context (Hetz et al., 2013). Interestingly, the ER chaperone immunoglobulin binding protein (BiP/GRP78), which is an early signal of ER stress activation, is required to give angiogenic potential to tumors (Dong et al., 2008), suggesting that ER stress might impact angiogenesis from the beginning of the response. However, in non-tumoral contexts, ER stress has been shown to impair cell migration and angiogenesis.

Tunicamycin is an antibiotic that inhibits synthesis of asparagine-linked glycoproteins (Takatsuki et al., 1971; Duksin and Bornstein, 1977) and is commonly used to induce ER stress in different *in vitro* and *in vivo* models. Promoting the accumulation of misfolded un-glycosylated proteins at the ER level, tunicamycin produces activation of all of the ER branches (Schroder and Kaufman, 2005). In support of the notion that ER stress impacts cell migration, early studies performed by Gipson et al. (1984) showed impaired epithelial sheet migration in the presence of tunicamycin. This study observed delayed wound healing in tunicamycin-exposed organotypic cultures of corneas (Gipson et al., 1984). Several years later, similar results were obtained *in vitro* with human epithelial airway cells (Dorscheid et al., 2001). In addition, recent observations in vascular smooth muscle cells show that tunicamycin activates the IRE1 and ATF6 pathways, imparing platelet-derived growth factor-induced *in vitro* migration (Yi et al., 2012). Similarly, neferine, an alkaloid used in cancer treatment, induces ER stress activation in an epithelial cell line, which produces concomitant inhibition of cell migration (Yoon et al., 2013).

Therefore, ER stress activation under resting or non-tumoral physiopathological conditions seems to impair collective cell migration, conversely to the tumoral context in which it seems to promote angiogenesis (Paridaens et al., 2014). This finding suggests that ER stress might play different roles in EC migration depending on the tissue environment.

### ROLE OF ER STRESS IN EC MIGRATION AND ANGIOGENESIS

**FIGURE 1:**
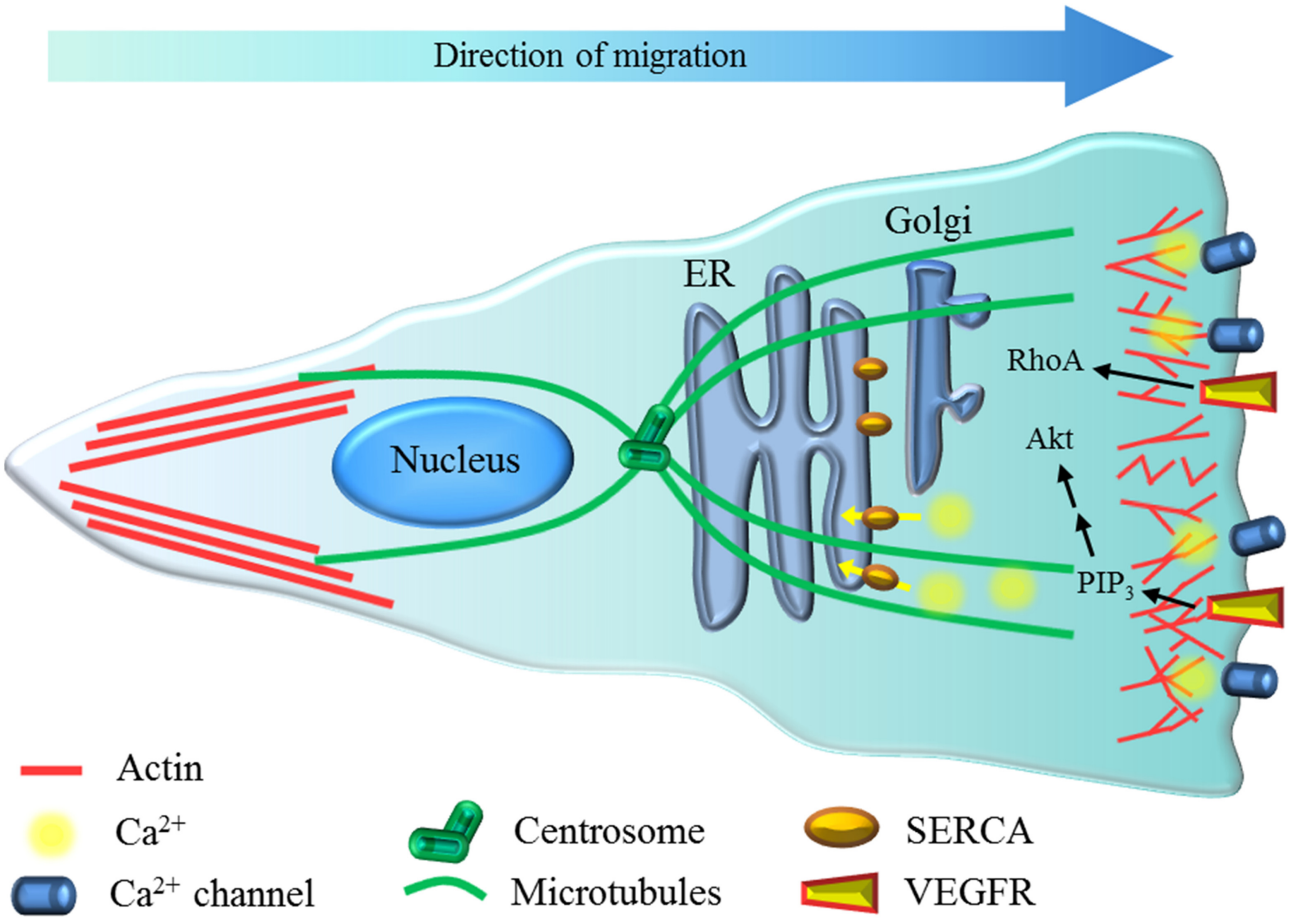
**Possible role of ER stress on EC migration.** Migrating EC have actin (red) dynamics at the front and also polarization of the centrosome and microtubules (green) and Golgi apparatus toward the leading edge. Ca^2+^ (yellow) dynamics at the front of the cell are mainly mediated by Ca^2+^ channels (i.e., Orai), and the sarco/endoplasmic reticulum Ca^2+^-ATPase pump (SERCA, orange) restores the free intracellular Ca^2+^ levels in the cytoplasm, allowing for sustained oscillations. At the rear of the cell, the formation of actin stress fibers is observed. Vascular endothelial growth factor receptor (VEGFR) activation induces RhoA and -through phosphatidylinositol (3,4,5)-triphosphate (PIP_3_) production- Akt activity, which contributes to cytoskeleton polarization and remodeling.

Collective EC migration is required as an initial event during angiogenesis. The EC migration process combines three different mechanisms: (1) chemotaxis, which is induced by soluble chemoattractants, (2) haptotaxis, which is mediated by chemoattractants bound to the substrate, and (3) mechanotaxis, which provides the mechanical forces to provide directionality (Lamalice et al., 2007). EC migrate according to the leader–follower model, in which a leader (or pioneer) cell with more protrusive and motile activity at the leading edge affects the signaling of the follower cells. Thus, the leader cell exerts mechanical pulling over the follower cells, providing the directionality of the sheet growth (Vitorino and Meyer, 2008; Rorth, 2009). At the cellular level, this process involves both actin and microtubule cytoskeleton rearrangements and changes in cell polarity (**Figure 1**) towards the edge of the monolayer (Etienne-Manneville, 2013).

While wound healing is commonly used to evaluate collective EC migration, tube formation in Matrigel has been used extensively to evaluate the angiogenic potential of these cells. These and other *in vitro* and *in vivo* models are used to evaluate different pro- or anti-angiogenic compounds (Lamalice et al., 2007; Aranda and Owen, 2009). As expected, VEGF is a potent chemoattractant for EC and contributes to angiogenesis (Lamalice et al., 2007). Interestingly, signaling of VEGF and other proteins involved in EC migration and angiogenesis is affected by ER stress (Paridaens et al., 2014).

Tunicamycin exposure is associated with impairment of both spontaneous and VEGF-induced migration of capillary EC, involving inhibition of VEGF signaling (Banerjee et al., 2011). Similarly, 2-DG reduces spontaneous collective migration, showing an anti-angiogenic effect on cultures of HUVEC through activation of ER stress (Merchan et al., 2010). Moreover, this study also shows that 2-DG has an anti-angiogenic effect *in vivo* (Merchan et al., 2010). In addition, treatment with neferine, another ER stress inducer, inhibits *in vitro* angiogenesis in HUVEC (Yoon et al., 2013). Similarly, acrolein, which is an unsaturated aldehyde known as an environmental pollutant and also found in some foods, induces ER stress in EC (Haberzettl et al., 2009), affecting both migration and angiogenesis (O’Toole et al., 2014). This study shows that acrolein inhibits wound healing and tube formation in HUVEC (O’Toole et al., 2014). Importantly, the same study evaluated Akt signaling after insulin exposure, showing that acrolein impairs insulin signaling (O’Toole et al., 2014), supporting the hypothesis that IR might be linked to distorted EC migration.

In contrast, MCPIP mediates cytokine-induced angiogenesis in HUVEC by up-regulation of ER stress markers (Roy and Kolattukudy, 2012). In addition, a recent study shows that VEGF might induce PERK and ATF6 signaling, which contribute to survival and migration of EC (Karali et al., 2014). These data suggest that in EC cells, the induction of ER stress might affect both migration and angiogenesis in a stimulus-dependent manner. Moreover, in a murine model it has been shown that IRE1 activity, which contributes to proper placental development, is required for development of ER stress during pregnancy (Iwawaki et al., 2009). This study shows that lack of IRE1 reduces VEGF receptor expression and is lethal (Iwawaki et al., 2009). These data suggest that in EC, induction of ER stress might affect both migration and angiogenesis in a stimulus-dependent manner.

One additional explanation of the different effects of ER stress on angiogenesis capacity could be related to the involvement of microenvironmental factors such as inflammatory mediators. For example, diabetic and other models of retinopathy are associated with distorted retinal angiogenesis, which has been related to ER stress (Salminen et al., 2010; Wang et al., 2012). In a murine model of type I diabetes, increased levels of VEGF and TNF-α were observed in the retina, which were correlated with increased ER stress markers of the PERK and IRE1 branches (Li et al., 2009). In addition, oxygen-induced retinopathy was associated with development of ER stress, in a similar way to that induced by tunicamycin (Li et al., 2009). Importantly, resveratrol and some of its derivatives (Tabata et al., 2007), which have anti-inflammatory effects and inhibit ER stress development (Zhang and Kaufman, 2008), prevent retinal vascular degeneration induced by tunicamycin or ischemia/reperfusion (Li et al., 2012). Taken together, these data suggest that ER stress contributes to angiogenesis and neovascularization *in vivo*. However, the target cells are not fully elucidated, and it is possible that the effect of ER stress inhibition might first impact immune cells, which through the release of inflammatory mediators might indirectly impact EC. Interestingly, several of these inflammatory mediators show altered levels during obesity (Snyder-Cappione and Nikolajczyk, 2013).

## EFFECTS OF MATERNAL OBESITY AND INSULIN RESISTANCE ON CELL MIGRATION

Altered nutritional state is becoming a relevant and growing public health issue globally [World Health Organ (WHO), 2003]. The relationship between obesity-induced chronic ER stress and IR has been well established in murine and human adipose tissues (Cnop et al., 2012; Flamment et al., 2012; Jung et al., 2013; Boden et al., 2014). Interestingly, it has been shown that the obesity-dependent induction of ER stress markers is reduced in human adipose tissue after weight loss, suggesting that body weight change constitutes an important factor that modulates the ER stress response (Gregor et al., 2009). During pregnancy, excessive gestational weight gain and MO have been associated with increased risk of maternal pathologies and detrimental long-term effects on fetal tissues, through a process known as intrauterine programming (McMillen and Robinson, 2005). Since HUVEC provides a useful model to study neonatal evidence of fetal EC programming under multiple pregnancy conditions, in this section we focus on different IR- and migration-associated proteins that might be distorted by MO.

### MO-RELATED FETAL PROGRAMMING

Obesity and overweight during pregnancy are well-recognized independent risk factors that contribute to the development of metabolic syndrome and several diet-related anomalies not only in the mother, but also in the fetus through fetal programming [American College of Obstetricians and Gynecologists (ACOG), 2005; Flenady et al., 2011; Triunfo and Lanzone, 2014]. This intrauterine programming can be observed as altered responses to physiological stimuli in HUVEC isolated from pathological pregnancies (Cheng et al., 2013; Krause et al., 2013). Indeed, it has been described that MO induces IR in fetuses *in utero* (Catalano et al., 2009), showing the relevance of metabolic fetal programming. Recently, it has been found that EC from obese adult subjects show ER stress (Kaplon et al., 2013), but it has not been determined whether MO induces these changes in fetal tissue. However, interesting recent evidence suggests that ER stress might be induced through fetal programming in animal models (Melo et al., 2014; Wu et al., 2014).

Using a murine model of MO, feeding dams a high-fat diet resulted in increased inflammation, ER stress markers, and IR in hypothalamic tissue of the MO offspring (post-natal day 28) compared to the control group (Melo et al., 2014). This study shows that lactation plays a major role in the development of ER stress (Melo et al., 2014). However, it was also noted that there was a significant increase in phosphorylation of eIF2α, downstream of PERK, in hypothalamic tissue at birth (day 0) of MO offspring (Melo et al., 2014), suggesting that at least the PERK ER stress branch is already activated during MO pregnancy.

In another study, using a similar model of diet-induced obesity, it was shown that MO offspring have increased ER stress and inflammatory markers compared to the control group (Wu et al., 2014). This study shows increased PERK and IRE1 activation in liver and adipose tissue of MO offspring at post-natal day 100 (Wu et al., 2014). Interestingly, treating dams during pregnancy and lactation with quercetin, which is an anti-inflammatory flavonoid (Indra et al., 2013) that inhibits ER stress (Suganya et al., 2014), prevented the development of ER stress in the offspring of MO pregnancies (Wu et al., 2014), suggesting that the development of ER in the offspring begins during pregnancy.

Altogether these data show that MO induces ER stress through fetal programming in murine models. Therefore, it is conceivable to suggest that MO in human pregnancies might produce a similar phenomenon.

### HOW MIGHT MO IMPACT HUVEC MIGRATION AND ANGIOGENESIS?

Fetal programming is known to occur during MO pregnancies; however one remaining question is how MO might mediate fetal EC migration and angiogenesis. First, human chorionic gonadotropin has been shown to increase the proliferation of HUVEC in the presence of various adipokines, such as IL 6, leptin, adiponectin, and TNF-α (Polec et al., 2014). Moreover, there is interesting evidence that placental tissue from women with MO shows altered expression of VEGF receptors (Saben et al., 2014). In fact, a very recent study showed that increased body mass index (BMI) was associated with the presence of angiogenic markers in placental tissue (Zera et al., 2014). This work demonstrates an inverse correlation between BMI and serum levels of sFlt-1, which is associated with a pro-angiogenic profile (Zera et al., 2014). The authors propose that this might be due to excessive fetal growth, which requires a bigger placental vascular bed (Zera et al., 2014). This distorted angiogenic profile during MO is also supported by evidence showing the predominance of non-branching angiogenesis observed in placental tissue of obese women (Dubova et al., 2011).

Considering that: (1) MO results in distorted angiogenesis; (2) obesity has been associated with ER stress and IR; and (3) IR-related proteins also play a role in cell migration (RhoA, Akt), we hypothesize the possible contribution of different IR-related and others proteins to the modulation of EC migration capacity in the context of MO-dependent ER stress (**Table 1**).

**Table 1:** Putative migration-related targets of ER stress signaling.

Target protein	Cell type	ER stress trigger	ER stress effect on target	Reference
RhoA	U87, HUVEC, HUVEC	↑IRE1, VEGF, ND	↓M ↑A, ↓M?	Dejeans et al. (2012), van Nieuw Amerongen et al. (2003), Song et al. (2012)
Spark	U87	↓IRE1	↑M ↑RhoA activity	Dejeans et al. (2012)
PI_3_K/Akt/GSK3β/β-Catenin/E2F2 via	HUVEC	VEGF	↑P ↑A	Zeng et al. (2013)
eNOS	HUVEC	CHOP-10	↓M?	Loinard et al. (2012)
HO-1	VSMC	Tunicamycin	↓M	Yi et al. (2012)
Tsp-1	Athymic Balb/c (nu/nu), CEC	Tunicamycin	↓A	Banerjee et al. (2011)
MCPIP	HUVEC	TNF-α, IL-1β, IL-8	↓A	Roy and Kolattukudy (2012)
Scrib	HUVEC	ND	↓A?	Michaelis et al. (2013)

*A, angiogenesis; M, migration; ND, not determined; P, proliferation.*

#### RhoA signaling

RhoA and its downstream signaling has been linked to IR because they have targets such as Akt and eNOS (Kanda et al., 2006; Nunes et al., 2010) but also play a relevant role in cell migration (Jaffe and Hall, 2005). As mentioned above, in 2D cultures, EC migrate according to the leader–follower model (Rorth, 2009). In HUVEC, fibroblastic growth factor-induced collective cell migration is commanded by proteins that regulate cell–cell interactions, cell density, individual cell migration, and directed cell migration (Vitorino and Meyer, 2008). In the same study, RhoA was found to contribute to collective cell migration of HUVEC, a finding corroborated later by other groups (Vitorino and Meyer, 2008; Povero et al., 2013). Moreover, the role of RhoA is also relevant in other cell types where it seems to be a typical feature of leader cells (Omelchenko et al., 2003; Jaffe and Hall, 2005; Rorth, 2009), because it contributes importantly to the mechanotaxis process (Reffay et al., 2014).

The contribution of RhoA to EC migration has been observed using a dominant-negative model and by its inhibition using ADP-ribosylation after bacterial toxin exposure. Both experimental conditions were associated with reduction of HUVEC migration (Aepfelbacher et al., 1997; Song et al., 2012) and angiogenesis (Povero et al., 2013). Previous studies suggest that RhoA mediates migration and VEGF-induced chemotaxis (van Nieuw Amerongen et al., 2003). However, interesting observations using a microfluidic device showed that RhoA contributes to HUVEC shear stress-induced mechanotaxis, although it does not affect VEGF-induced filopodia formation (Song et al., 2012). The explanation for these differential effects may also rely on the EC culture type studied; for example, RhoA contributes to VEGF-induced migration and angiogenesis of human foreskin microvascular EC (van Nieuw Amerongen et al., 2003). Thus, it is possible to suggest that RhoA contributes to migration in a stimulus- and cell type-dependent manner.

**FIGURE 2:**
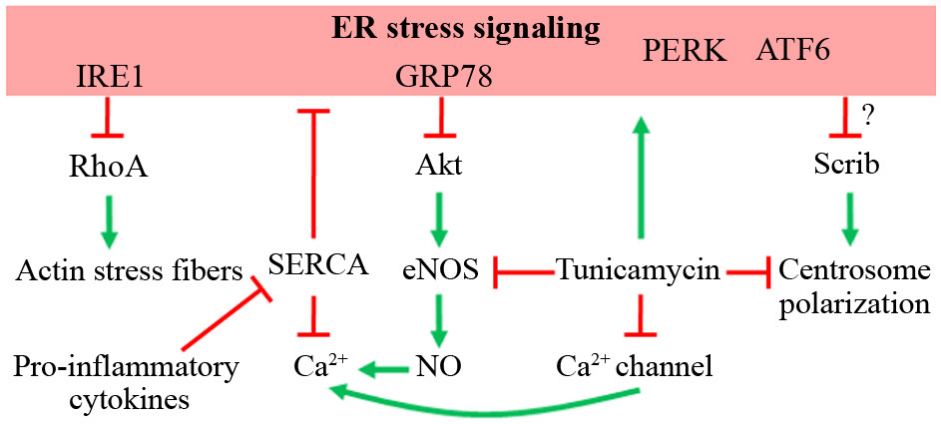
**Possible role of ER stress on EC migration.** The effects of ER stress signaling on different proteins that command cellular migration is depicted, as well as the effect of tunicamycin, an ER stress inducer. Red lines indicate inhibition or down-regulation of the target. Green arrows indicate activation or up-regulation of the target.

As mentioned above, ER stress is linked to cell migration. Supporting this notion, ER stress is associated with IRE1, which acts as an upstream protein of RhoA signaling (Dejeans et al., 2012). In a RhoA-dependent manner, cancer cells expressing a dominant-negative IRE1 protein show increased adhesion, impaired migration, and a reduced proliferation rate, but no change in invasive properties (Dejeans et al., 2012). As expected, RhoA inhibition restores the phenotype in IRE1 dominant-negative expressing cells (Dejeans et al., 2012). Therefore, as IRE1-lacking cells show over-activation of RhoA, it is possible to hypothesize that MO-induced ER stress, which increases IRE1 activity, might reduce RhoA activity, impairing EC migration (**Figure 2**).

#### Akt signaling

The Akt gene encodes three isoforms (Akt1-3). In EC, Akt activation is related to several signaling cascades, such as the insulin pathway, eNOS activation, cell survival, and migration (Shiojima and Walsh, 2002). In EC, it has been suggested that Akt3 contributes to cell migration (Vitorino and Meyer, 2008). However, Akt involvement in cellular migration and angiogenesis depends on the tissue context and simultaneously activated signaling (Somanath et al., 2006). For instance, Akt3 has defective signaling in muscle cells from obese insulin-resistant subjects (Brozinick et al., 2003), potentially affecting EC migration response downstream of the insulin receptors (Guo, 2014). In addition, extensive evidence has shown a potential link between Akt and ER stress signaling (Appenzeller-Herzog and Hall, 2012). Interestingly, BiP/GRP78 suppresses the Ser473 phosphorylation of Akt by direct interaction, which might prevent accessibility for activating kinases (Yung et al., 2011). Moreover, this study shows that ER stress activates or inhibits Akt signaling depending on the magnitude or severity of this response (Yung et al., 2011). On the other hand, VEGF has been related to induction of the IRE1 branch of ER stress in HUVEC (Zeng et al., 2013). VEGF exposure was associated with IRE1-dependent splicing of XBP1 and activation of Akt/GSK signaling, which is required for the proliferation and angiogenesis induced by this growth factor (Zeng et al., 2013).

Downstream to Akt activation, the production of NO by eNOS has been linked in EC migration, because pharmacological inhibition of eNOS reduces *in vitro* migration capacity (Murohara et al., 1999; Lamalice et al., 2007). Moreover, aortic EC from eNOS-lacking mice have impaired *in vitro* and *in vivo* angiogenesis (Lee et al., 1999; Lamalice et al., 2007). Tunicamycin-induced ER stress reduces eNOS levels in mouse coronary artery EC (Galan et al., 2014). Accordingly, homocysteine-induced ER stress through C/EBP CHOP-10 signaling inhibits eNOS signaling in HUVEC (Loinard et al., 2012). As mentioned above, fetuses from pregnancies with MO develop IR (Catalano et al., 2009), which is maintained through childhood (Bruyndonckx et al., 2013). Since eNOS activation is regulated by insulin stimulation in EC, eNOS signaling could be altered in HUVEC from MO pregnancies as an outcome of fetal programming, as occurs in other maternal pathologies (Farías et al., 2006, 2010; Leiva et al., 2011; Westermeier et al., 2011). If the latter effectively occurs, HUVEC derived from MO pregnancies might have impaired Akt/eNOS signaling and migration and/or angiogenic capacity (**Figure 2**).

#### Soluble mediators and Ca^2+^ signaling

Pro-inflammatory mediators such as cytokines and adipokines are increased during MO (Catalano et al., 2009) and have detrimental effects on EC migration. Leptin is an adipokine with pro-angiogenic effects that induces HUVEC migration after activation of Akt and eNOS (Goetze et al., 2002). However, leptin exposure is not associated with significant effects on tube formation *in vitro* (Dubois et al., 2013). In contrast, adiponectin inhibits HUVEC migration in a wound-healing assay and also inhibits tube formation *in vitro* (Dubois et al., 2013), showing an opposite effect to that of leptin. In fact, leptin signaling is impaired by ER stress, which also contributes to leptin resistance (Hosoi et al., 2008; Ozcan et al., 2009). Conversely, adiponectin has been linked to inhibition of ER stress (Boddu et al., 2014).

Pro-inflammatory cytokines, such as TNF-α, IL-1β, and IL-8, have a pro-angiogenic effect (Dinarello, 2007). These cytokines exert this effect by up-regulation of MCPIP, which is required to induce angiogenesis *in vitro* by increasing ER stress (Roy and Kolattukudy, 2012), suggesting an association between cytokines and ER stress. Interestingly, most if not all cytokines regulate free intracellular Ca^2+^ levels in cells, providing another possible link between these soluble mediators and the development of ER stress, as we discuss next.

Ca^2+^ signaling is one of the most important players during cell migration (Wei et al., 2012). Ca^2+^ channel-dependent calcium dynamics are observed at the leading edge in migrating cells (Wei et al., 2009). Consequently, during HUVEC migration, a polarized generation of PIP_3_ is found at the front of the migrating cells, which further increases the Ca^2+^ influx, allowing cytoskeleton rearrangements required for motility (Tsai and Meyer, 2012; Tsai et al., 2014). Simultaneously, an increase in the extrusion of Ca^2+^ towards the extracellular milieu is observed, hence maintaining the Ca^2+^ dynamics at the front (Tsai et al., 2014), showing that a fine-tuning of Ca^2+^ signaling is required for HUVEC migration. In this context, tunicamycin links ER stress with Ca^2+^ signaling because this antibiotic induces distorted function of Ca^2+^-channels (Czyz et al., 2009). In addition, NO produced by eNOS contributes to Ca^2+^ dynamics (Huang et al., 2013), suggesting that deficient eNOS signaling induced by ER stress might affect Ca^2+^ signaling and hence cell migration. Interestingly, pro-inflammatory cytokines IL-1β and IFN-γ down-regulate the SERCA and increase ER stress markers in pancreatic β-cells (Cardozo et al., 2005). The possibility of a similar mechanism occurring in EC is interesting, because it would unveil the mechanisms by which cytokines might affect EC migration (**Figure 2**).

#### Cell polarity

During cell migration, a reorientation of several cellular structures occurs in a process called polarization (Rorth, 2009). One of the intracellular features exhibited by migrating EC is the polarization of the centrosome toward the direction of movement of the endothelial sheet (**Figure 1**; Gotlieb et al., 1981; Rorth, 2009; Etienne-Manneville, 2013). Moreover, microtubule-binding drugs that inhibit HUVEC migration exert this blockade effect by avoiding centrosome repositioning (Hotchkiss et al., 2002; Kamath et al., 2014). Therefore, ER stress might affect cellular polarization and hence impair cell migration.

One of the candidates potentially affected by ER stress is Scrib, which mediates chemotaxis-dependent, but not spontaneous, cell migration (**Figure 2**) and *in vitro* and *in vivo* angiogenesis (Michaelis et al., 2013). This protein contributes to cytoskeletal rearrangements and Golgi apparatus polarization toward the leading edge in wound-healing assays (Michaelis et al., 2013). Whether a similar distortion occurs with nuclei and/or mitochondrial and/or lysosomal reorientation (Rorth, 2009; Friedl et al., 2011; Etienne-Manneville, 2013; da Silva et al., 2014) has not yet studied. Thus, cell polarity-related proteins might be affected by ER stress and thus impair proper organelle and centrosome polarization.

## CONCLUDING REMARKS AND PERSPECTIVES

Endothelial cell migration relies on tightly regulated signaling cascades that are activated by various stimuli. Adequate signaling events are required for proper remodeling of vessels during angiogenesis, and distorted intracellular cross-talk among the involved pathways would result in vascular dysfunction. We focus on the potential involvement of two main mechanisms of disease observed in obesity, ER stress, and IR. Interestingly, ER stress might impact EC migration and hence angiogenesis in different ways. Here, we summarize the current knowledge about how ER stress might provoke alterations in EC migration capacity and propose new targets (**Figure 1**). Specifically, both ER stress and IR might affect the coordination of endothelial chemotaxis, haptotaxis, mechanotaxis, Ca^2+^ signaling, and cell polarity modulation, which are key steps associated with EC migration. Better understanding of these processes regarding the physiopathological mechanism underlying ER stress might provide new perspectives in the design of therapeutic targets.

Different ER stress stimuli and micro-environmental contexts play major roles in regulation of EC migration, as well as the timing of stimulation signals and the magnitude of ER stress activation. For example, a physiological role of ER stress has been shown during pregnancy, where it is required for placenta development (Iwawaki et al., 2009), but it still is unknown whether its overactivation under pathological conditions remains favorable or becomes detrimental. On the other hand, intracellular cascades associated with IR development may be also associated with impaired EC migration capacity. To further address these research topics, new models of *in vivo* and * in vitro* analysis are required. An interesting approach recently validated the use of rat mesenteric EC to evaluate angiogenesis, because these cells exhibit the same behavior as HUVEC during migration and angiogenesis (Mansouri et al., 2013). Another approach proposes 3D culture of adipocytes and HUVEC in microspheres, in an attempt to mimic adipose tissue (Yao et al., 2013). The zebrafish, a well-established model to evaluate migration and angiogenesis, has been recently used to evaluate the role of Akt and ER stress pathways (Lu et al., 2014). Furthermore, the chick embryo chorioallantonic membrane assay might be used to evaluate the impact of ER stress on EC migration and angiogenesis, in a way similar to its current use in evaluating the anti-angiogenic potential of different compounds (Lange et al., 2014). Taken together, these models might provide new tools for studying EC migration and angiogenesis during obesity-induced ER stress and IR.

Considering the state of knowledge, we propose that acute and chronic ER stress might induce different effects on EC migration. In addition, as observed in tumoral versus non-tumoral environments, ER stress might promote or impair EC migration and angiogenesis, respectively. Finally, based on the hypothesis of intrauterine programming during pregnancies affected by adverse conditions and the induction of ER stress and IR in the presence of obesity, we suggest that MO might induce fetal ER stress and IR, two intracellular mechanisms associated with altered EC migration and hence distorted angiogenesis in offspring endothelium.

## Conflict of Interest Statement

The authors declare that the research was conducted in the absence of any commercial or financial relationships that could be construed as a potential conflict of interest.

## ABBREVIATIONS

2-DG: 2-deoxy-D-glucose
ATF6: activating transcription factor 6
BiP/GRP78: immunoglobulin binding protein
BMI: body mass index
CHOP-10: C/EBP homologous protein-10
EC: endothelial cell
eIF2α: eukaryotic translational initiation factor 2α
eNOS: endothelial nitric oxide synthase
ER: endoplasmic reticulum
GSK: Akt/glycogen synthase kinase
HUVECs: human umbilical vein endothelial cells
IFN-γ: interferon γ
IL: interleukin
IR: insulin resistance
IRE1: inositol-requiring enzyme 1α
MCPIP: monocyte chemotactic protein-induced protein
MO: maternal obesity
PERK: PKR-like eukaryotic initiation factor 2α kinase
PIP_3_: phosphatidylinositol (3,4,5)-trisphosphate
RhoA: Ras homolog family member A
Scrib: scribbled planar cell polarity protein
SERCA: sarco/endoplasmic reticulum Ca^2+^-ATPase pump
sFlt-1: soluble fms-like tyrosine kinase-1
TNF-α: tumor necrosis factor α
UPR: unfolded protein response
VEGF: vascular endothelial growth factor
XBP1: X-box binding protein 1
